# Prevalence and predictors of atherosclerotic renal artery stenosis in hypertensive patients undergoing simultaneous coronary and renal artery angiography; a cross-sectional study

**DOI:** 10.15171/jrip.2016.08

**Published:** 2016-02-18

**Authors:** Babak Payami, Mehrian Jafarizade, Seyed Seifollah Beladi Mousavi, Shahab-Aldin Sattari, Forough Nokhostin

**Affiliations:** ^1^Department of Cardiology, Faculty of Medicine, Ahvaz Jundishapur University of Medical Sciences, Ahvaz, Iran; ^2^Chronic Renal Failure Research Center, Ahvaz Jundishapur University of Medical Sciences, Ahvaz, Iran; ^3^Faculty of Medicine, Ahvaz Jundishapur University of Medical Sciences, Ahvaz, Iran

**Keywords:** Renal artery stenosis, Hypertension, Coronary angiography

## Abstract

**Introduction:** According to the non-specific presentation of atherosclerotic renal artery stenosis (ARAS), this disease is usually an under-diagnosed in clinical conditions.

**Objectives:** The aim of the presence study was to evaluate the prevalence of renal artery stenosis (RAS) and its related risk factors in hypertensive patients undergoing coronary angiography.

**Patients and Methods:** In a cross-sectional study, between March 2009 and October 2010, all of hypertensive patients candidate for diagnostic cardiac catheterization, underwent nonselective renal angiography before completion of their coronary angiography procedure. A standardized questionnaire was used to collect demographics, cardiac history, indications for cardiac catheterization and angiographic data. The degree of ARAS was estimated visually by skilled cardiologist. Narrowing greater than 50% of the arterial lumen considered as arterial stenosis. Data was analyzed by SPSS version 19, and by chi-square test and logistic regression model.

**Results:** In overall 274 patients with mean age of 60.75 ± 10.92 years 108 (39.4%) were male and 166 (60.61%) were female. The prevalence of ARAS calculated 18.2%. According to the present study, heart failure and smoking were predictors of ARAS. However, old age, gender, diabetes mellitus, hyperlipidemia and family history of cardiovascular disease were not clinical predictors of significant ARAS in hypertensive patients, candidate for coronary angiography.

**Conclusion:** According to present data, we suggest to consider renal artery angiography in combination with coronary artery angiography especially in hypertensive patients who are smoker or individuals who have heart failure.

**Implication for health policy/practice/research/medical education:**

Considering that atherosclerotic renal artery stenosis (ARAS) is prevalent in hypertensive patients undergoing coronary angiography, thus screening for early diagnosis of ARAS is crucial for saving renal function and prevents others complication and prolongs patients life, all by timely intervention. Hence, we suggest to conduct a renal angiography in conjunction with coronary catheterization in hypertensive patients, especially in smoker hypertensive cases and hypertensive individuals who suffer from heart failure.

## Introduction

Renal artery stenosis (RAS) is defined as narrowing of the renal artery lumen (1). The most common etiology of RAS is atherosclerosis, which involves the main renal artery (2).

RAS is the primary cause for nearly 6% of the end-stage renal disease (ESRD) patients which is a life-threatening diseases with significant complication (3-7). In addition RAS is the etiology for 20% of dialysis individuals who are older than 50 years (1).

RAS is a leading factor of secondary hypertension, ischemic nephropathy and ESRD. Additionally, it is a progressive disease that, renal artery perfusion slowly declines and eventually renal function and structure deteriorates (2).

Individuals with atherosclerotic renal artery stenosis (ARAS) are frequently asymptomatic and have no characteristic laboratory test, making its timely diagnosis a major clinical problem. On the other hand early diagnosis of ARAS is of a great importance while timely intervention can improve clinical outcome (1). ARAS and coronary artery disease (CAD) are two manifestations of a same pathogenesis which is atherosclerosis. Thus, it is not an unusual event that a patient with CAD suffers from ARAS and conversely (8).

Existence of ARAS worsens the CAD course and prognosis (2). On the other hand CAD is one of the most important reasons of death in ARAS patients (9). The prevalence of RAS in individuals with suspected CAD who underwent coronary angiography was reported 11.3% to 39% by some studies (1). 1%-5% of individuals with blood hypertension (HTN) suffer from ARAS, while it is the most etiology of secondary HTN (2). Due to high coexistence of ARAS and CAD in cases with angiography diagnosed CAD (10) it has been advised renal angiography be performed in conjunction with coronary angiography (9).

## Objectives

Considering that ARAS is prevalent in hypertensive patients and since there is no data about the prevalence of ARAS in our region and due to the fact that identifying predictive risk factor for ARAS in individuals who are suspected for CAD is of great importance, hence, this study provides a golden opportunity for timely intervention which can improve ARAS clinical course and prognosis. Therefore, this study aimed to estimate the prevalence of ARAS in hypertensive patients who referred to our hospital, heart center and identify predictive factor for ARAS in above mentioned population.

## Patients and Methods

### Study patients

This is a prospective cross-sectional study which performed between March 2009 and October 2010 in Ahvaz, Emam hospital.

The inclusion criterion was any hypertensive patient who was suspected for CAD and referred to the hospital, heart center. The exclusion criteria were the followings: 1) weight more than 120 kg; 2) presence of acute renal failure and 3) history of contrast media sensitivity.

Thirty-eight persons from 312 individuals were excluded from the study due to of exclusion criteria and 274 cases enrolled the study.

Informed consent was obtained from all candidates before entering the study.

All patients fulfilled a questionnaire consisted of demographic data (including age and sex) and history of smoking, diabetes, hyperlipidemia, heart failure, HTN, renal failure, familial history of cardiovascular disease (CVD).

### Determination of hypertension

Presence of HTN was determined by either the history of HTN and taking antihypertensive agents or blood pressure above140/90 mm Hg on two serial physical exams.

Selected individuals underwent renal angiography after the completion of their coronary angiography by femoral approach, using a pig tail catheter and injecting 30 cc non-ionizing contrast agent (ominopaque 350, visipaque 320) with the speed of 15 cc/s. Narrowing greater than 50% of the coronary and renal artery lumen were considered as CAD and RAS respectively. CAD and RAS were reported by two experienced interventional cardiologists.

### Ethical issues

1) The research followed the tenets of the Declaration of Helsinki; 2) informed consent was obtained, and they were free to leave the study at any time; and 3) research was approved by the ethical committee of Jundishapur University of Medical Sciences, Ahvaz, Iran.

### Statistical analysis

Data were analyzed with chi-square test and logistic regression model by SPSS version 19. A *P* value below 0.05 were considered significant.

## Results

Of total 312 hypertensive patients, 274 patients enrolled in the study. The enrolled cases underwent renal angiography just after coronary angiography. The mean±SD of age of patients who enrolled in the study were 60.75±10.92 (ranging from 29 to 88 years). 108 (39.4%) were male and 166 (60.6%) were female.

Table 1 demonstrates the frequency and distribution of enrolled patients from the point view of their risk factors including smoking, diabetes mellitus, hyperlipidemia, renal failure, heart failure and familial history of CVD.

**Table 1:** Frequency of patients from the point view of their risk factors

**Risk factor**	**Yes ** **No. (%)**	**No ** **No. (%)**
Smoking	37 (13.5)	137 (86.5)
Diabetes mellitus	98 (35.76)	176 (64.24)
Hyperlipidemia	126 (45.98)	148 (54.02)
Heart failure	122 (44.52)	152 (55.48)
Renal failure	23 (8.39)	251 (91.96)
Familial history of CVD	39 (14.23)	135 (85.77)

Abbreviation: CVD‏, cardiovascular disease.

Based on the definition of RAS in our study, 50 patients (18.2%) identified as RAS cases. Of 50 patients, 42 individuals (14.16%) had unilateral RAS and 9 patients (3.24%) suffered from bilateral RAS.

**Figure 1:**
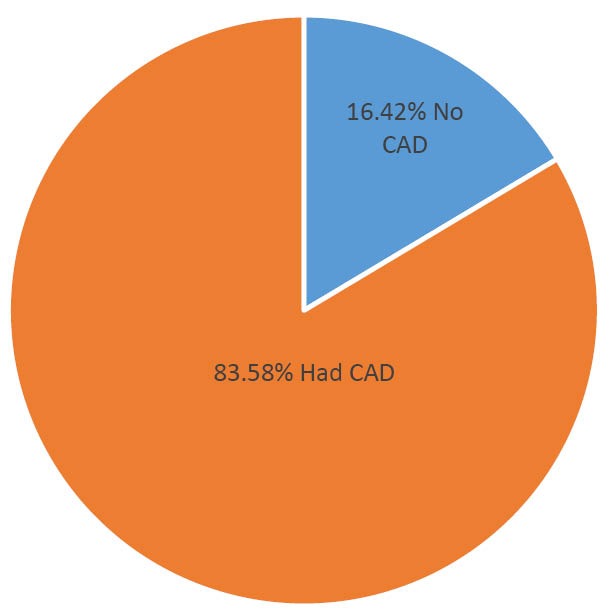
Distribution of CAD patients in whole study population.

**Figure 2:**
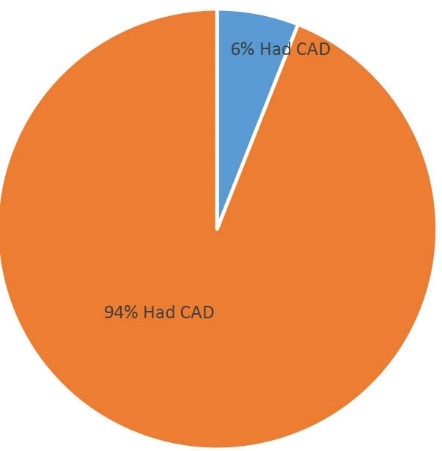
Distribution of CAD patients in RAS group.

Our findings from coronary angiography are shown in Figures 1 and 2. Mono-vessel, two vessels, and three vessels disease were found in 18.31%, 20.42% and 44.85% respectively (Figure 1). Mono vessel, two vessels, and three vessel involvements were identified 24%, 26% and 44% respectively (Figure 2).

Table 2 represents baseline clinical features of the study population in RAS and non-RAS groups and possible predictive risk factor for ARAS. Smoking and heart failure in hypertensive case can predict the existence of ARAS significantly.

**Table 2:** Baseline clinical features of the study population

	**No-RASn=224 (81.8%)**	**RASn= 50 (18.2%)**	***P*** ** value**
Age (years)	59.8±11.1	64±10.1	0.731
Sex ‏ (%)			0.091
Male	90 (40.2)	18 (36)	
Female	134 (59.8)	32 (64)	
Smoker (%)			0.001
Yes	27 (11.8)	10 (20)	
No	197 (88.2)	40 (80)	
Diabetes mellitus (%)			0.465
Yes	82 (36.6)	16 (32)	
No	142 (63.4)	34 (68)	
Heart failure (%)			0.022
Yes	98 (30.3)	24 (48)	
No	154 (69.7)	26 (52)	
Hyperlipidemia (%)			0.375
Yes	106 (47.3)	20 (40)	
No	118 (52.7)	30 (60)	
Renal failure (%)			0.336
Yes	18 (8)	5 (10)	
No	206 (92)	45 (90)	
Family history of CAD (%)			0.638
Yes	33 (14.7)	6 (12)	
No	191 (85.3)	44 (88)	

## Discussion

In this study, we investigated hypertensive patients who were suspected for CAD and underwent simultaneous coronary and renal artery angiography and found that the prevalence of ARAS in this population was 18.2% which was nearly high. 14.99% of the study population had unilateral ARAS while 3.24% of them suffered from bilateral ARAS.

In the study conducted by Rokni et al (between October 2009 and July 2011) at Tehran heart center, the prevalence of ARAS was reported as 40% in their study group (10). This high prevalence in this study is due to the fact that most of their cases who underwent renal angiography suffered from resistant HTN and renal dysfunction; whereas, just 8.3% of our study population had renal failure and most of our cases had controlled HTN.

In another study conducted by Rimoldi et al, the prevalence of ARAS in hypertensive patients who referred for coronary angiography was reported 8% (11). On the other hand the study conducted by Shah et al in Peshawar (12) and the study performed by Yamashita et al (13), the prevalence of ARAS was estimated 13%.

These differences in the reported prevalence from various studies is partly related to the demographic differences including difference in participants mean age and existence or absence of underlying diseases which worsen atherosclerosis. Accordingly, this discrepancy between results is partly attributed to the ethnical, regional and lifestyle pattern differences. High prevalence of atherosclerotic risk factors in our society and inactivity and low activity in large spectrum of people as well as atherogenic diet can justify this high prevalence of ARAS which reported by our study.

In this cross-sectional study we assessed the relationship between ARAS and the following risk factors, including age, sex, diabetes mellitus, hyperlipidemia, smoking, renal failure, heart failure, and familial history of CVD. We found that smoking and/or presence of heart failure could predict ARAS in hypertensive patients significantly (*P* value: 0.001; 0.022 respectively).

Smoking as predictive factor of ARAS is in contrast with some others study findings (13-19). This controversy can be explained by the coexistence of other diseases of above mentioned study participants, while all of individuals who enrolled in our study were hypertensive and therefore had at least one risk factor for atherosclerosis. Above controversy can be justified more by the difference in pack-years cigarettes smoking among population in which our study population had greater cigarette pack-year rate than others study participants probably.

Based on our study findings, the presence of heart failure in hypertensive patients can predict ARAS. This finding is in concordance with finding of a study performed by Tumelero et al which they concluded the presence of a strong relationship between left ventricular dysfunction and ARAS (20).

This fact can be clarified in this way that heart failure syndrome is a chronic disease in nature (regardless of some clinical state which called acute heart failure) and occurs years after an index event (mostly an atherosclerotic induced cardiovascular event).

Hence, the presence of heart failure syndrome in hypertensive patients represents the several years of atherosclerosis risk factors existence in such patients which leads to systemic arterial atherosclerotic stenosis potentially.

As with the finding of other studies (21-25), we found no significant relationship between diabetes, hyperlipidemia, familial history of CVD and ARAS existence, thus these findings are another stamps of verification that hypertensive patients with above mentioned risk factor who are suspected for CAD and must be evaluated angiographically from the point view of coronary vasculature should not underwent simultaneous renal artery angiography.

In this study we found no relationship between sex and ARAS which is in contrast with some other studies (23). They concluded that there is a relationship between female sex and the presence of ARAS. Wang et al explained this relationship by the fact that their female participants were older than male cases (25). This finding should be clarified by the future studies, since we expect that atherosclerosis onset is earlier in men individuals and its severity to be greater in male sex apparently.

Based on our results, no relationship between age and ARAS was existed, which is in contrast with some other studies(18-22). This result should further tested with larger samples.

## Conclusion

To the best of our knowledge, this study is the first report which demonstrates the predictive role of smoking and heart failure for ARAS in hypertensive individuals. We suggest further studies in order to reassess these predictive roles in future.

Considering that ARAS is relevant in hypertensive patients undergoing coronary angiography (one case from each five individuals, nearly) and screening for early diagnosis of ARAS is crucial for saving renal function and prevents others complication and prolongs patients life, all by timely intervention, we suggest renal angiography in conjunction with coronary catheterization in hypertensive patients, especially in smoker hypertensive cases and hypertensive individuals suffer from heart failure.

## Limitations of the study

The limitations of our study was small sample size and short duration of investigation.

## Acknowledgments

The authors wish to thank the dean of cardiology center trans­plantation of Ahvaz Jundishapur University of Medical Sciences for his help in data collection.

## Authors’ contribution

All authors contributed to manuscript equally.

## Conflicts of interest

The authors declared no competing interests.

## Ethical considerations

Ethical issues (including plagiarism, data fabrication, double publication) have been completely observed by the authors.

## Funding/Support

This paper is extracted from thesis of Mehrian Jafarizade (Thesis number: CRD-9303) and financial support was provided by the Chronic Renal Failure Research Center of Ahvaz Jundishapur University of Medical Sciences.
